# Predictive accuracy of boosted regression model in estimating risk of venous thromboembolism following minimally invasive radical surgery in pharmacological prophylaxis-naïve men with prostate cancer

**DOI:** 10.1186/s12957-023-03170-y

**Published:** 2024-02-23

**Authors:** Chie Hui Leong, Sushil Rodrigues Ranjan, Anna Javed, Basim S.O. Alsaedi, Ghulam Nabi

**Affiliations:** 1 Academic Urology Unit, Division of Imaging Sciences and Technology, School of Medicine, University of Dundee, Ninewells Hospital, Dundee, DD1 9SY UK; 2 Department of Pharmacology, AIIMS, Vijaypur, Jammu India; 3 Department of Statistics, University of Tabuk, 71491 Tabuk, Saudi Arabia

## Abstract

**Background:**

Venous thromboembolism (VTE) is a potentially life-threatening but preventable complication after urological surgery. Physicians are faced with the challenges of weighing the risks and benefits of thromboprophylaxis given scanty evidence for or against and practice variation worldwide.

**Objective:**

The primary objective of the study was to explore the possibility of a risk-stratified approach for thromboembolism prophylaxis following radical prostatectomy.

**Design, setting, and participants:**

A prospective database was accessed to cross-link venous thromboembolism events in 522 men who underwent minimally invasive prostatectomy between February 2010 and October 2021. A deterministic data linkage method was used to record events through electronic systems. Community Health Index (CHI) numbers were used to identify patients via electronic health records. Patient demographics and clinical characteristics such as age, comorbidities, Gleason staging, and readmission details accrued.

**Outcomes:**

VTE within 90 days and development of a risk-stratified scoring system. All statistical analysis was performed using R-Statistical Software and the risk of VTE within 90 days of surgery was estimated via gradient-boosting decision trees (BRT) model.

**Results and limitations:**

1.1% (6/522) of patients developed deep vein thrombosis or pulmonary embolism within 3 months post-minimally invasive prostatectomy. Statistical analysis demonstrated a significant difference in the body mass index (*p* = 0.016), duration of hospital stay (*p* < 0.001), and number of readmissions (*p* = 0.036) between patients who developed VTE versus patients who did not develop VTE. BRT analysis found 8 variables that demonstrated relative importance in predicting VTE. The receiver operating curves (ROC) were constructed to assess the discrimination power of a new model. The model showed an AUC of 0.97 (95% confidence intervals [CI]: 0.945,0.999). For predicting VTE, a single-center study is a limitation.

**Conclusions:**

The incidence of VTE post-minimally invasive prostatectomy in men who did not receive prophylaxis with low molecular weight heparin is low (1.1%). The proposed risk-scoring system may aid in the identification of higher-risk patients for thromboprophylaxis.

**Patient summary:**

In this report, we looked at the outcomes of venous thromboembolism following minimally invasive radical prostatectomy for prostate cancer in consecutive men. We developed a new scoring system using advanced statistical analysis. We conclude that the VTE risk is very low and our model, if applied, can risk stratify men for the development of VTE following radical surgery for prostate cancer.

## Introduction

Venous thromboembolism (VTE) is a significant postoperative complication characterized by thrombi formation in the deep veins of the body. It most commonly occurs in the legs, a condition known as deep vein thrombosis (DVT). Up to 10% of untreated DVTs can lead to pulmonary embolism (PE) where the thrombus dislodges from the vein wall and travels to the pulmonary arteries [1, 2]. Interestingly, the incidence of VTEs is found higher in cancer patients as cancer cells can stimulate the clotting cascade directly by generating thrombin, and indirectly by activating platelets, endothelial cells, and mononuclear cells to express procoagulants [3]. When assessing the risk of VTE in cancer patients, prostate cancer patients generally have a lower VTE risk as compared to other solid cancers such as lung and pancreatic cancer. This risk is variable depending on the type of procedure (open radical prostatectomy 1.0–15.7%, minimally invasive 0.4–6.0%, and robotic 0.2–3.7%) and is increased with the extent of pelvic lymph node dissection (PLND) involved [4].

The negative sequelae of VTEs include the risk of post-thrombotic syndrome and the risk of recurrences (up to 25% for DVT) [5]. DVT and PE are associated with significant mortality with reported rates of mortality being 6% and 12%, respectively. They also contribute to the millions of cost burden placed on the National Health Service (NHS) annually [5, 6].

Most patients are given intermittent pneumatic compression devices to reduce the risk of VTE, and many health centers have also implemented the use of thromboprophylaxis for patients who have no contraindications [7]. However, the lack of condition-specific evidence on the risk–benefit of pharmacological prophylaxis following radical prostatectomy especially in low-risk cases, and the conflicting guidelines have contributed to large variations in the use of thromboprophylaxis within and between countries [8]. Recent European Association of Urology (EAU) guidelines have made recommendations for future research to focus on comprehensive characterization of prospective study cohorts that include careful documentation of follow-up time, clear record of venous thromboembolism episodes, and bleeding and to address the unclear duration for prophylaxis [9].

The focus of this present study is to fill in the knowledge gap in some of these areas using a prospectively collected cohort of men who had radical prostate surgery in a defined geographical area with a population migration of less than 1%. Follow-up was available for each patient without pharmacological prophylaxis after minimally invasive radical prostatectomy through record-linkage methodology. Furthermore, we aimed to develop a risk-stratified approach (score) to guide clinical practice and to identify patients at risk of VTE so that prophylactic treatments could be tailored to this group. This has been carried out using boosted regression tree analysis. Later approaches have been used to provide a combination of optimal statistical predictive performance and reliable identification of relevant variables by fitting different models.

## Patients and methods

A prospective database was maintained for all the consecutive men undergoing minimally invasive radical prostatectomy for clinically localized prostate cancer from February 2010 to October 2021. The study had prior institutional review board approvals in place through the Caldicott process. All men were followed up using a protocol, and their records were linked using a unique 10-character numeric community health index (CHI). The CHI is a registration number for all patients and residents in the region. The number is used to provide universal health coverage to all the residents and record their episodes of care in primary, secondary, and social care. All medical services, including free prescriptions during a hospital visit, are state-funded as a part of a legal guarantee of universal healthcare coverage. Table 1 shows the demographic details of the cohort as identified from the database. Using CHI, we linked electronic healthcare records of the cohort to identify re-admission, including prescriptions and imaging data. This was a deterministic data linkage study whereby the unique Community Health Index (CHI) numbers were used to identify events via electronic health records.

**Table 1:** Comparison of sociodemographics and clinical features between patients who developed VTE against patients who did not develop VTE post-surgery

Variables	Non-VTE patients (*n* = 634)	VTE patients (*n* = 6)	*P* value
**Median age**	66 (range 42–79)	64 (range 56–69)	
**Body mass index**			
Underweight	1 (0.2%)	0 (0.0%)	**0.016**
Normal	159 (25.1%)	0 (0.0%)	
Overweight	284 (44.8%)	2 (33.3%)	
Obese	182 (28.7%)	4 (66.7%)	
Unknown	8 (1.3%)	0 (0.0%)	
**Operative time (min)**	Mean 180 (130–246)	Mean 170 (160–200)	0.621
**Prostate gland size (gram on MRI)**	Mean 40 (range 25–250)	Mean 56 (40–123)	0.460
**Clinical stage**			
T1	2(0.4%)	0	0.632
T2	599 (94.4%)	4	
T3	33 (5.2%)	2	
**Gleason staging**			
6	53 (8.4%)	0 (0.0%)	0.580
7	418 (65.9%)	5 (83.3%)	
8	61 (9.6)	1 (16.7%)	
9	95 (15.0%)	0 (0.0%)	
Unknown	7 (1.1%)	0 (0.0%)	
**Comorbidities**			
< 2	220 (34.7%)	1 (16.7%)	0.325
≥ 2	414 (65.3%)	5 (83.3%)	
**Previous VTE**			
Yes	16 (2.5%)	1 (16.7%)	0.055
No	618 (97.5%)	5 (83.3%)	
**Extended pelvic lymph node dissection**			
Yes	549 (86.6%)	5 (83.3%)	0.454
No	47 (7.4%)	0 (0.0%)	
Unknown	38 (6.0%)	1 (16.7%)	
**Immediate postoperative complications**			
Yes	95 (15.0%)	3 (50.0%)	0.055
No	500 (79.0%)	3 (50.0%)	
Unknown	38 (6.0%)	0 (0.0%)	
**Duration of hospital stay**			
< 5 days	471 (74.3%)	3 (50.0%)	**< 0.001**
≥ 5 days	126 (19.9%)	3 (50.0%)	
Unknown	37 (5.8%)	0 (0.0%)	
**Number of readmissions**			
0	431 (68.0%)	2 (33.3%)	**0.036**
≥ 1	196 (30.9%)	4 (66.7%)	
Unknown	7 (1.1%)	0 (0.0%)	
**Living status**			
Dead	35 (5.5%)	2 (33.3%)	**0.010**
Alive	599 (94.5%)	4 (66.7%)	

Sociodemographic variables, clinical features, and readmission details such as age, prostate gland size, cTstage, operative time, body mass index (BMI), comorbidities, previous history of VTE, number, and causes of readmissions were recorded. The primary outcome of the study was to identify men who developed VTE during a follow-up of 90 days of surgery. The secondary outcome of the study was to develop a scoring system to identify men at higher risk of VTE following minimally invasive radical surgery for clinically localized prostate cancer.

### Developing risk stratified scoring system from variables

Clinical variables were converted into factor variables and risk points were ascribed depending on co-efficient in logistic regression. The total score for each of the above variables was used to represent the prediction of re-admission probability. Descriptive and inferential data analyses using the recorded data were performed using the *R* test statistical software.

**Fig. 1:**
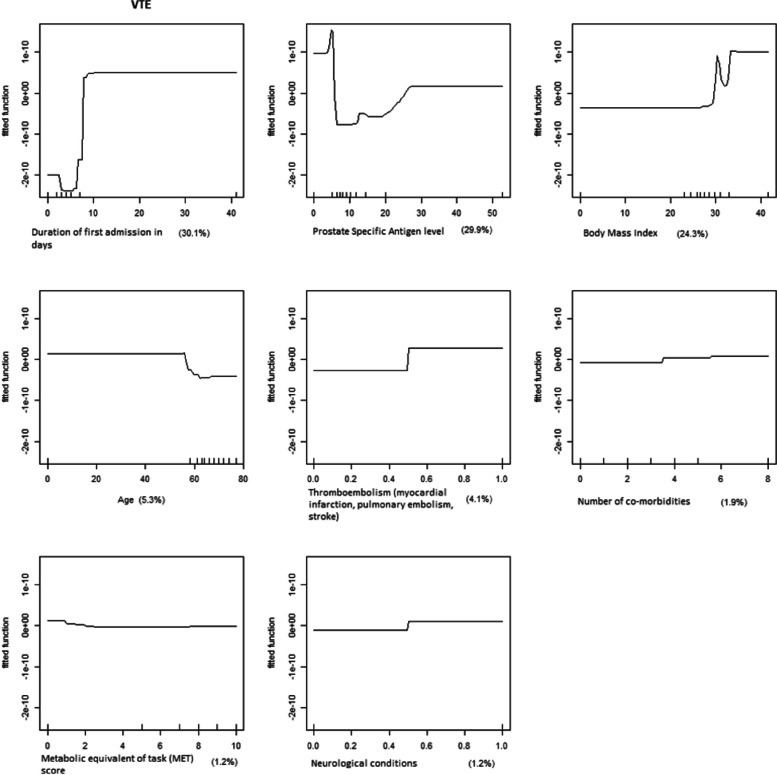
Graphs from boosted regression tree analysis showing variables of relative importance in the proposed risk-stratified scoring system

A boosted regression tree (BRT) model approach was applied to provide an improved regression model for the VTE dataset. The BRT is an approach whereby insights and techniques from both the statistical and maximum likelihood traditions are drawn. The choice of BRT was based on the fact that it utilizes the boosting technique that combines multiple tree models to optimize the predictive performance [10]. To obtain specific cut-off conditions for each important variable, the authors have used adaptive (manual) selection based on graphs from the BRT analysis (Fig. 1).

**Fig. 2:**
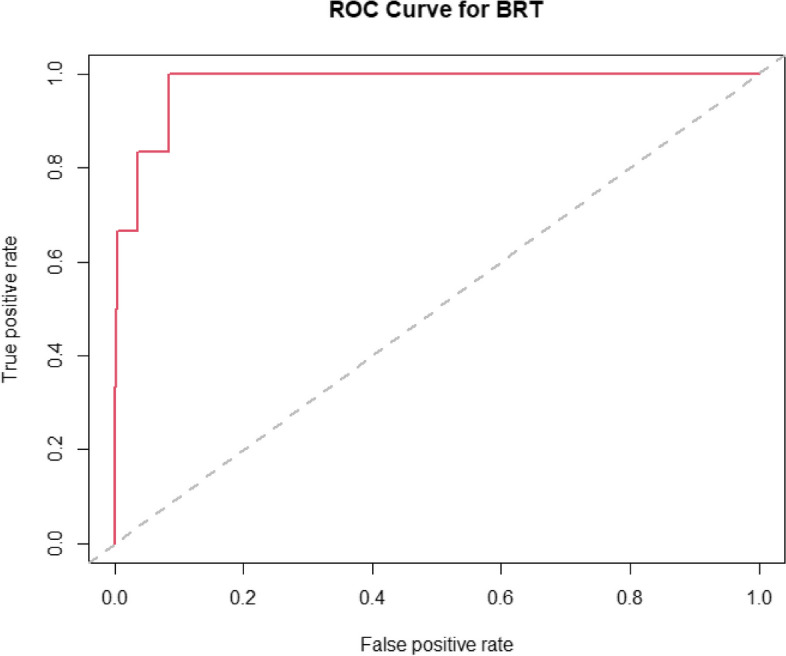
A receiver operator characteristic (ROC) curve

A receiver operator characteristic (ROC) curve was created to assess the performance of the model (Fig. 2) focusing on sensitivity, specificity, positive predictive value, and negative predictive value.

Based on the BRT analysis, variables that demonstrated relative importance in predicting VTE post-minimally invasive prostatectomy were identified. These variables were used to construct the risk-stratified scoring system. Variables with the highest relative importance weighed a higher score and vice versa. Variables with relative importance of less than 1 were omitted from the risk-stratified scoring system.

Specific scores for each variable were given to patients who satisfy the condition. Finally, the overall score for each patient is the summation of scores across all the important variables.

### Determining the cut-off point for the risk-stratified scoring system

A cut-off point refers to an optimal threshold value of the risk-stratified scoring system distribution that identifies and differentiates high-risk from low-risk patients. Hereby, we proposed a new simulation-based method to obtain a cut-off point using the above risk-stratified scoring system. Data of VTE-only patients was resampled to produce 10,000 different datasets of size 5. The risk-stratified scoring system criteria were used to score the resampled patients. For every sampled dataset, the lower quantile, 0.025, of the distribution in the score system was calculated. The cut-off point was then obtained by calculating the distribution mean of this score system. The proposed method was compared with other cut-off point methods namely Youden index and bootstrapping methods through the R packages cut-point and Optimal cut-point, respectively.

## Results

### Sociodemographics and clinical features of the VTE group

A total of 640 prostate cancer patients underwent minimally invasive prostatectomy within the study period. Among them, 522 (81.6%, 522/640) patients did not receive thromboprophylaxis. Six (1.1%, 6/522) of these patients were found to have developed pulmonary embolism within 3 months postoperatively.

The median age of the VTE group was 64 years (range 56–69) whereby 5 (83.3%, 5/6) had at least 2 co-morbidities, and all had a BMI > 24.9. A total of 5 (83.3%, 5/6) patients had a Gleason scoring of 7, and 5 (83.3%, 5/6) patients had extended pelvic lymph node dissection. Three (50.0%, 3/6) patients had immediate postoperative complications, and 3 (50.0%, 3/6) patients required 5 or more days of hospital stay. Four (66.7%, 4/6) patients were readmitted at least once 3 months post-surgery.

Statistical tests have demonstrated a significant difference in the body mass index (*p* = 0.016), duration of hospital stay *(p* < 0.001), number of readmissions (*p* = 0.036), and living status (*p* = 0.010) between patients who developed VTE versus patients who did not develop VTE. Complete data is presented in Table 1.

### Developing the risk-stratified scoring system

Results from the BRT analysis found 8 variables that demonstrated relative importance in predicting VTE (Table 2).

**Table 2:** Boosted regression tree (BRT) results showing variables of relative importance in predicting VTE and their respective conditions from manual adaptation

Risk factors	Relative importance	Scoring	Conditions
**Duration of first admission**	30.130091	5	> 8 = 5; 0
**Prostate-specific antigen**	29.935732	4	> 10 = 4; 0
**Body mass index**	24.340618	4	> 30 = 4; 0
**Age**	5.259972	2	> 65 = 2; 0
**Clotting diseases**	4.0502079	2	> 0 = 2; 0
**Number of comorbidities**	1.9164005	2	> 3 = 2; 0
**METS score**	1.2396053	2	> 3 = 2; 0
**Neurological diseases**	1.1763001	2	> 0 = 2; 0
**Gleason score**		1	> 6 = 1; 0

The receiver operating curves (ROC) were constructed to assess the discrimination power of the new model. Figure 2 showed an AUC of 0.97 (95% confidence intervals [CI]: 0.945, 0.999). The cut-off limits for each variable were manually selected based on graphs from BRT analysis (Fig. 1).

The top 3 factors accounting for the highest relative importance were duration of first admission, level of prostate-specific antigen (PSA), and body mass index (BMI). Variables of relative importance above 30.0, 20.0, 10.0, and 1.0 were scored 5, 4, 3, and 2, respectively, on the risk-stratified scoring system. Gleason staging was not found to be of relative importance from this dataset. The authors have, however, included one point for Gleason scoring above 6 given its evidence in predicting VTE in the literature [11, 12]. The average score for our VTE patients following the above risk-stratified scoring system was 10.6 while the average score for non-VTE patients was 7.5.

### Determining the cut-off point for the risk-stratified scoring system

The total score of this proposed risk-stratified scoring system for a patient considering all variables was 24. Given the relatively small study population, and to get statistically significant cut-off scores for high-risk/low-risk patients, a simulated dataset was produced. On the dataset, a 10,000 resampling from patients with VTE was used. Notably, the results remained stable when the resampled size was increased, from 5 to 100. The cut-off score based on our proposed simulation methodology was 6.1.

The results obtained on the simulated dataset were calibrated using the real-life study population. Therein, of the six VTE-positive patients, 5 had a score ≥ 6 (more precisely, the scores were (scores of 6, 9, 14, 15, 15), indicating high-risk patients (equal to positive control). However, one of the VTE-positive patients had a score of 5, which indicates a low-risk patient. The cut-off score of 6.1 calculated on the simulated dataset should be thus lowered to ≥ 5 to include all the 6 VTE-positive patients from the study population.

Patients above this cut-off could be considered for thromboprophylaxis post-radical prostatectomy whilst patients scoring < 5 could be considered as low-risk patients. In total, there were 102 (102/522; 19.5%) patients who had scored < 5 based on our proposed scoring system.

## Discussion

Postoperative VTE has been a major concern amongst surgeons for many decades, as has been the risk of bleeding postoperatively. To reduce VTE risk, some surgeons prescribe pharmacological thromboprophylaxis while others opt for only mechanical compressions and early mobilization (Ref). This variation in practice is largely due to challenges in weighing the benefits of reducing VTE and the risk of major bleeding with pharmacological thromboprophylaxis [4]. To achieve a trade-off, various attempts have been made to develop risk-prediction models for predicting the development of VTE in surgical patients. These models include the Wells score and the Caprini score for stratifying risks for VTE, which are already validated and utilized in clinical practice [13, 14]. However, there are currently no radical prostatectomy procedure-specific scoring systems, most certainly for minimally invasive approaches.

The practice of VTE prophylaxis following urological procedures is debated across the globe. In a survey conducted by Gavin et al., only 24% of urologists from the USA had reported to have prescribed thromboprophylaxis as compared to higher percentages (100% and 50%, respectively) in Britain and Ireland [15]. In this present study, 28 days of prophylactic Dalteparin was prescribed starting September 2019, to all patients without contraindications upon discharge. However, no pharmacological prophylaxis was practiced between 2010 and 2019.

The 30-day postoperative incidence of VTE is very variable among urological surgeries. Reported rates are 5.5%, 1.9%, and 1.1% for radical cystectomy (113/2,065), radical nephrectomy (52/4568), and radical prostatectomy (178/16,484), respectively [16]. Our study shows a relatively consistent result of 1.1% (6/522) of VTE within 3 months post-radical prostatectomy. In this study based on the National Surgical Quality Improvement Program database in the USA, Alberts et al. reported that 82.6% (147/178) of patients who developed VTE after radical prostatectomy, VTE only developed after discharge from hospital [16]. Similarly, 66.7% (4/6) of our VTE incidences also occurred only after discharge. This highlights the burden of VTE beyond the time of discharge; thus, the identification of high-risk patients is crucial to guide the extended duration of thromboprophylaxis in outpatient settings.

The main question that stems from this study is whether thromboprophylaxis should be offered to all patients with post-radical prostatectomy. From the results of our study, the authors feel that thromboprophylaxis should be guided by risk stratification rather than the religious use of thromboprophylaxis for all patients due to the following reasons: (1) only 1.1% (6/522) of our study population developed VTE within 3 months postoperatively, (2) 98.9% (516/522) of patients who were not given prophylaxis never developed VTE within 3 months postoperatively, and (3) self-administration of thromboprophylaxis at home may be unnecessary and challenging both mentally and physically for some patients. To support this, Koya et al. have also reported a low 0.21% of VTE incidences over 12 years in 1364 patients who underwent retropubic radical prostatectomy which again questioned the routine use of VTE prophylaxis postoperatively. Two other articles have also concluded no significant reduction in VTE when thromboprophylaxis was offered [14, 17].

In a study from China, Cheng et al. reported a very high incidence (11.4%, 40/351) of VTE in men undergoing robotic-assisted radical prostatectomy procedures. This is much higher than the reported literature from the western parts of the world. One of the reasons could be advanced disease as the mean PSA in those with VTE was 46.97 ng/ml, required a longer operation time, and extended lymph node dissection. Nevertheless, the study reported two new models and a nomogram to predict the risk of VTE. Our approach to data analysis has been different and steered towards establishing a scoring system.

There is an urgent need to produce a risk-scoring system to identify specific patients requiring thromboprophylaxis. The Caprini model has a universal standard for predicting VTE and was introduced to identify patients at risk. However, this scoring system may be more complex and time-consuming to use as it considers over 30 indicators to assess the VTE risk. Moreover, the uniqueness of prostate cancer also warrants a scoring system specific to itself [14]. Hence, we have used our dataset to produce a risk-stratified scoring system unique to prostate cancer men who underwent minimally invasive prostatectomy. Similar to the present study, the ROC reported by Cheng et al. (reference) showed an AUC of 0.988 (95% confidence intervals [CI], 0.977–1.000) for Model B and 0.957 (95% CI 0.928–0.985) for Model A. The AUC for the model used in the present study was 0.97 (95% confidence intervals [CI]: 0.945, 0.999) which is comparable to Cheng et al. but much better than the model proposed by Caprini et al. with an AUC of 0.807 (95% CI 0.700–0.914).

In view of the findings from the present study data, we are proposing a risk-stratified approach to guide thromboprophylaxis specifically for post-minimally invasive prostatectomy patients. We have found the duration of hospital stay, PSA, and BMI had the highest predictive value of VTE. A cut-off point of ≥ 5/24 was obtained for high-risk patients, and 19.5% (102/522) of patients who scored < 5 were low-risk. This shows that thromboprophylaxis could potentially be avoided safely in the 102 low-risk patients who did not develop VTE. The development of this new scoring system may therefore be helpful in avoiding unnecessary thromboprophylaxis in low-risk patients.

### Strengths and limitations

This study has limitations including it being a single center with no external validation. The extent to which this approach can be extended into more complex situations is unclear and found to be worth future research efforts. It should be noted that it is likely that a larger study population may help in improving cut-off scores definition. We have attempted to compensate for low numbers by simulation of the dataset based on the real-life prospectively collected episodes of care. The strengths of the present study lie within its prospective design where a cohort of patients was observed with adequate follow-up post-procedure. Data collection also covers all episodes of care recorded in the system linked to each patient through Community-Health-Index numbers unique to each patient. Our results represent the data of a defined geographical area, and they are obtained based on robust modeling of our dataset including the re-sampling of data. We did consider validating our model using previously reported risk models such as the PADUA risk scoring system [18]; however, significant differences in the population of patient, methodology, and non-procedure/condition-specific nature of the model precluded the possibility of any useful information with additive value to the study.

## Conclusion

Venous thromboembolism (VTE) post-minimally invasive prostatectomy in pharmacological prophylaxis naive patients is low. Boosted regression tree (BRT) analysis-based scoring system further risk-stratified men where the risk is extremely low and prophylaxis is not needed. The proposed risk-stratified approach could add some insight to the literature for the development of a future guideline.

## Data Availability

All the data is available for a third party on request.
